# Spontaneous Hyperventilation Is Common in Patients with Spontaneous Cerebellar Hemorrhage, and Its Severity Is Associated with Outcome

**DOI:** 10.3390/jcm11195564

**Published:** 2022-09-22

**Authors:** Zhuangzhuang Miao, Huajian Wang, Zhi Cai, Jin Lei, Xueyan Wan, Yu Li, Junwen Wang, Kai Zhao, Hongquan Niu, Ting Lei

**Affiliations:** 1 Department of Neurosurgery, Tongji Hospital Affiliated to Tongji Medical College of Huazhong University of Science & Technology, Wuhan 430030, China; 2 Department of Neurosurgery, Wuhan Fourth Hospital, Puai Hospital, Wuhan 430030, China

**Keywords:** spontaneous hyperventilation, cerebellar hematoma, arterial blood gas test, outcome

## Abstract

Background: The spontaneous hyperventilation (SHV) accompanying spontaneous cerebellar hemorrhage has yet to attract a sufficient amount of attention. This study aimed to analyze the incidence of SHV in spontaneous cerebellar hemorrhage patients and its risk factors as well as its association with the outcome. Methods: We retrospectively reviewed the medical records of all spontaneous cerebellar hemorrhage patients who underwent surgical treatment at Tongji Hospital from July 2018 to December 2020. Arterial blood gas (ABG) test results and clinical characteristics, including demographics, comorbidities, imaging features, laboratory tests, and therapy choices, were collected. The Glasgow Outcome Scale was used to assess the outcome at two weeks and six months after admission. Results: A total of 147 patients were included, and of these patients 44.9% had spontaneous hyperventilation. Hypertension (OR, 3.175; CI, 1.332–7.569), usage of sedation drugs (OR, 3.693; CI, 1.0563–8.724), and hypernatremia (OR, 2.803; CI, 1.070–7.340) seemed to positively correlate to SHV occurrence. Hematoma removal had an inverse association with SHV (OR, 0.176; CI, 0.068–0.460). Patients with poor and good outcomes had significant differences in pH, PaCO_2_, and HCO_3_^−^ values, and the severity of SHV was associated with the PaCO_2_ level. Conclusions: Spontaneous hyperventilation is common in patients with spontaneous cerebellar hemorrhage, and its severity is associated with the outcome.

## 1. Introduction

Carbon dioxide is considered to be a powerful modulator of the cerebral vasculature [1,2,3]. Hypocapnia is induced to lower the intracranial pressure (ICP) by decreasing the cerebral blood volume (CBV) via cerebral arterial vasoconstriction [4]. Experiments have shown that the cerebral blood flow (CBF) decreases by approximately 3% per mmHg change in PaCO_2_ (60 to 20 mmHg) in patients with a traumatic brain injury (TBI) [5]. Thus, intentional hyperventilation, which induces controlled hypocapnia, was once thought to be a treatment for intracranial hypertension (ICH) in patients with a traumatic brain injury (TBI) [6].

However, not all hyperventilation is intentional. Central spontaneous hyperventilation (SHV) has also been observed in patients with a brain injury [7], subarachnoid hemorrhage [8,9], and brain tumors [10,11,12]. Generally, diagnostic criteria for SHV include low arterial PaCO_2_, high arterial PaO_2_, and high arterial pH that persists during sleep without the impact of drug or metabolic causes [13]. However, significant reductions in CBF induced by reductions in PaCO_2_ may also lead to hypoxia of the brain parenchyma [14]. Thus, the potential benefits of hyperventilation for ICH could be offset by these reductions in brain tissue oxygenation [15,16].

Spontaneous cerebellar hemorrhage, as the most serious and least treatable type of stroke, accounts for approximately 10% of all spontaneous intracerebral hemorrhages (ICHs), with a 30-day mortality varying from 30% to 50% [17,18,19]. Due to the narrow confines of the posterior fossa, obstructive hydrocephalus or a local mass effect on the brainstem or transforamen magna herniation may occur quickly in the early stage of cerebellar hemorrhage [20].

In clinical practice, spontaneous hyperventilation is often observed in cerebellar hemorrhage patients and, as this manifestation has not attracted a sufficient amount of attention, few studies have focused on it. Therefore, we attempted to study whether spontaneous hyperventilation is a common manifestation in cerebellar hemorrhage patients and its risk factors as well as its association with the prognosis.

## 2. Materials and Methods

### 2.1. Patient Selection and Ethical Approval

All patients with spontaneous cerebellar hemorrhage admitted to the neurointensive care unit (NICU) at Tongji Hospital, Tongji Medical College, Huazhong University of Science and Technology, China between July 2018 and December 2020 were screened in this retrospective study. Spontaneous cerebellar hemorrhage was diagnosed by a computed tomography (CT) scan and satisfied the following criteria: the patient had no history of trauma, single fourth ventricular or brainstem hemorrhage was excluded, multiple localized intracerebral hemorrhage involving the cerebellum was excluded, and hemorrhage caused by a clear etiology, such as cerebellar arteriovenous malformations and brain tumors, was excluded.

All patients were treated in accordance with international guidelines [20] and underwent surgical treatment. Care was provided by an experienced multidisciplinary critical care team, typically for at least one week.

The inclusion criteria were as follows: age ≥18 years, onset with only spontaneous cerebellar hemorrhage, and an arterial blood gas (ABG) test at least once during NICU hospitalization for at least one week. Patients were excluded if they had invalid ABG test results.

All procedures performed in studies involving human participants were performed in accordance with the ethical standards of the Medical Ethics Committee of Tongji Hospital, Tongji Medical College, Huazhong University of Science and Technology, the 1964 Declaration of Helsinki and its later amendments, or comparable ethical standards. The requirement for patient consent was waived as this retrospective study had no effect on the rights, health, and privacy of the subjects. The risk to the subjects of this study was not greater than the minimum risk.

### 2.2. ABG Test Results and Definition of SHV

Patients took the ABG test every two days or depending on the actual needs. ABG tests were performed when the body temperature was normal and before or at least 30 min after nursing care or other pain management. If the patient was mechanically ventilated, pressure support ventilation (PSV) was preferred since it improves patient comfort and ventilator synchrony, and ABG tests were used to determine the effectiveness of the PSV settings. As the patient triggers each breath in PSV mode, the patient has more control over the flow delivery and respiratory rate. There tends to be less ventilator desynchrony from patient-triggered breaths during inspiration or passive exhalation and less voluntary movement of the diaphragm compared with delivered breaths [21]. ABG tests were performed when the patient breathed above the set respiratory rate of the pressure support (PS) mode in order to exclude interference from iatrogenic hyperventilation as much as possible [22]. All patients had spontaneous breathing, and endotracheal intubation was performed to ensure airway patency depending on the patient’s respiratory status. For patients who need long-term mechanical ventilation, a minimal amount of sedation, such as dexmedetomidine, which does not significantly inhibit respiration [23], may be provided in order to prevent ventilator desynchrony and patient discomfort. For patients who breathe comfortably in the PSV mode, the amount of sedation can often be decreased, allowing for more interaction while awake and participation in physical therapy [24]. The respirator’s ventilation mode and parameters were determined by the attending neurointensivist based on the needs of the patient, and conflicts between humans and machines were avoided as much as possible.

SHV was defined by the presence of at least one ABG with both PaCO_2_ < 35 mmHg and pH > 7.45 and was divided into three groups according to the PaCO_2_ results: mild (30 mmHg ≤ PaCO_2_ < 35 mmHg), moderate (25 mmHg ≤ PaCO_2_ < 30 mmHg), and severe (PaCO_2_ < 25 mmHg) [4,9].

The parameters of the ABG results were further analyzed and defined as follows: acidemia for pH < 7.35, alkalemia for pH > 7.45, hypocapnia for PaCO_2_ < 35 mmHg, and hypercapnia for PaCO_2_ > 50 mmHg. The complex etiology of the respiratory system and metabolism were not considered in all results.

### 2.3. Data Collection

Demographic data (sex and age) and medical history (comorbidities, including hypertension, diabetes mellitus, heart dysfunction, lung dysfunction, and others, and time from onset to admission) for each patient were retrieved by interviews with the patient and/or family on admission. Information on the location and volume of the hematoma, subarachnoid hemorrhage, and hydrocephalus was obtained from computed tomography (CT) scan imaging data. The rest of the information, including laboratory test results (routine analysis of blood, liver function, and electrolyte level), physical examination results (state of consciousness, GCS, etc.), surgical methods, and other information (mechanical ventilation, sedation drugs, fever, and pneumonia), was collected from the medical record system.

Pneumonia was defined by the decision of the treating physician to complete a full antibiotic treatment course based on a chest X-ray, culture results, and clinical symptoms. Fever was treated aggressively with antipyretics and surface cooling devices. Abnormal test results were defined as follows: hypoproteinemia for albumin < 35 g/L, hypernatremia for blood sodium > 145 mmol/L, hyponatremia for blood sodium < 135 mmol/L, hypokalemia for blood potassium < 3.5 mmol/L, hypocalcemia for blood calcium < 1.1 mmol/L, hyperglycemia for fasting blood glucose > 6.1 mmol/L, and anemia for hemoglobin < 120 g/L.

The Glasgow Outcome Scale (GOS) was used to assess g/L neurologic functional outcomes at 2 weeks and 6 months in the short term and long term, respectively. The GOS score is a 5-point scale, where 1 = death, 2 = persistent vegetative state, 3 = severe disability, 4 = moderate disability, and 5 = good recovery [25]. GOS scores of 1–3 were divided into groups of poor outcomes, while scores of 4–5 were considered good outcomes.

### 2.4. Statistical Analysis

Numerical variables are reported as counts and proportions (%). The χ^2^ test or Fisher’s exact test (if the sample size was less than 5) was used to compare differences in nominal variables between the groups. A normality test (the Kolmogorov–Smirnov Z test) and a variance homogeneity test were performed for continuous variables, which are reported as the mean and standard deviation (SD) if they obeyed a normal distribution or the median and interquartile range (IQR) if they did not. Student’s t test was used for normally distributed variables to compare the difference between two groups, and the Mann–Whitney U test was used for non-normally distributed variables.

Variables that were considered clinically relevant to SHV occurrence or that showed significant differences between the SHV group and the non-SHV group were entered into a multivariate logistic regression model. Variables for inclusion were carefully chosen, given the number of events available, to ensure that the final model would be parsimonious. The final results for each variable are expressed as the odds ratio (OR) and 95% confidence interval (95% CI).

All statistical analyses were performed with IBM SPSS Statistics version 23.0 (Chicago, IL, USA) For all tests, *p* < 0.05 was considered significant.

## 3. Results

### 3.1. SHV Occurrence in Patients

A total of 192 patients were admitted to our neurointensive care unit during the study period, and 147 of these patients satisfied the criteria for inclusion. Sixteen patients were excluded because they had invalid ABG test results, such as missing values or substandard sampling. Nine patients were hospitalized in the NICU for less than one week. Nine patients were younger than 18 years old. Eleven patients were lost by follow-up.

**Figure 1:**
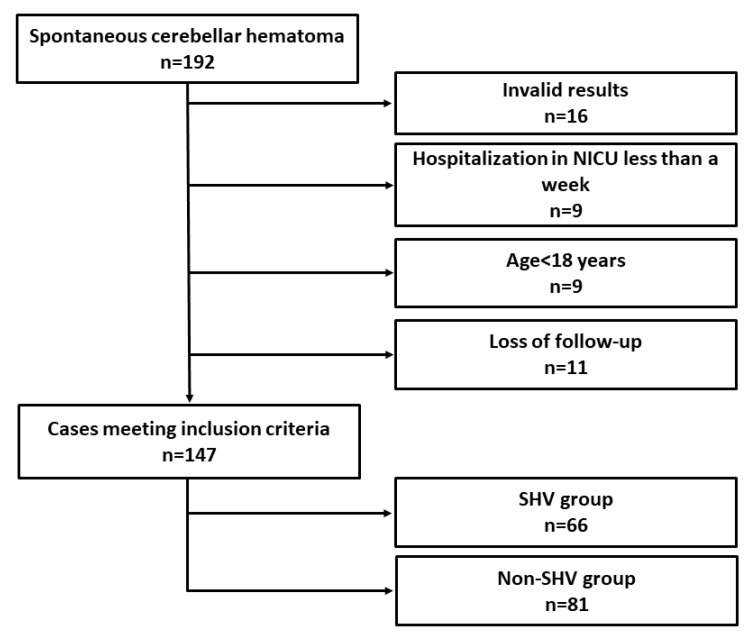
Flowchart of study cases satisfying the inclusion criteria. NICU, neurointensive care unit; SHV, spontaneous hyperventilation.

Of the 147 patients who were eligible for the study, 66 (44.9%) had ABG test results with both PaCO_2_ < 35 mmHg and pH > 7.45 at least once and were classified as the spontaneous hyperventilation group. The remaining 81 (55.1%) patients without spontaneous hyperventilation were treated as the nonspontaneous hyperventilation group (Figure 1).

### 3.2. Clinical Characteristics

The clinical characteristics of the study population are described in Table 1. Groups with and without spontaneous hyperventilation were compared. There was a lower proportion of male patients in the SHV group compared with the non-SHV group (48.5% vs. 67.9%, *p* < 0.05). The median age was 59 years in the SHV group and 58 years in the non-SHV group (*p* > 0.05). A higher incidence of hypertension was observed in the SHV group compared with the non-SHV group (69.7% vs. 48.1%, *p* < 0.01). However, the incidences of other comorbidities, such as diabetes mellitus (21.2% vs. 11.1%), heart dysfunction (7.6% vs. 9.9%), lung dysfunction (9.1% vs. 11.1%), and others (10.6% vs. 13.6%), showed no significant difference (*p* > 0.05). Hematomas located on the unilateral side or middle line were not different between the SHV group and the non-SHV group (54/12 vs. 67/14, *p* > 0.05), whereas their volume was greater in the SHV group (19.8 ± 8.3 vs. 16.8 ± 8.7, *p* < 0.05). More than half of the patients in the two groups had subarachnoid hemorrhage (51.5% vs. 63.0%, *p* > 0.05), and similar proportions of hydrocephalus occurred (13.6% vs. 16.0%, *p* > 0.05).

Notably, although the median GCS score assessed at admission appeared to be the same in the two groups, the statistical analysis still showed a significant difference (*p* < 0.05), especially for the proportion of GCS < 6 (31.8% vs. 8.6%, *p* = 0.001). However, the times from onset to admission (12.0 (7.0, 24.0) vs. 12.0 (6.0, 24.0), *p* > 0.05) and from admission to surgery (10.0 (4.0, 20.3) vs. 12.0 (7.0, 20.5), *p* > 0.05) were not different, and the abnormal laboratory tests at admission included hypoproteinemia (12.1% vs. 18.5%, *p* > 0.05), hypernatremia (21.2% vs. 13.6%, *p* > 0.05), hypokalemia (18.2% vs. 19.8%, *p* > 0.05), hypocalcemia (9.1% vs. 8.6%, *p* > 0.05), hyperglycemia (81.8% vs. 86.4%, *p* > 0.05), and anemia (40.9% vs. 33.3%, *p* > 0.05).

The proportion of patients who underwent hematoma removal was slightly lower in the SHV group compared with the non-SHV group (57.6% vs. 80.2%, *p* < 0.05). In contrast, the proportions of hematoma removal with decompressive craniectomy (54.5% vs. 58.0%, *p* > 0.05), external ventricular drainage (45.5% vs. 42.0%, *p* > 0.05), and tracheotomy (37.9% vs. 40.7%, *p* > 0.05) were similar in both groups.

After the operation, the number of days of mechanical ventilation (4.0 (3.0, 5.0) vs. 3.2 (1.8, 4.1), *p* = 0.001), usage of sedation drugs (69.7% vs. 43.2%, *p* = 0.001), and number of days of fever (3.5 (2.0, 6.0) vs. 3.0 (1.0, 4.0), *p* < 0.05) were significantly higher in the SHV group. The incidence of pneumonia (60.6% vs. 67.9%, *p* > 0.05) and postoperative laboratory tests, including hypoproteinemia (84.8% vs. 92.6%, *p* > 0.05), hyponatremia (13.6% vs. 12.3%, *p* > 0.05), hypokalemia (53.0% vs. 38.3%, *p* > 0.05), hypocalcemia (69.7% vs. 82.7%, *p* > 0.05), hyperglycemia (83.3% vs. 84.0%, *p* > 0.05), and anemia (66.7% vs. 75.3%, *p* > 0.05), were not significantly different except for hypernatremia (42.4% vs. 19.8%, *p* < 0.05).

In addition, patients with poor outcomes assessed by the GOS score accounted for a higher proportion in the SHV group in both the short term (*p* < 0.05) and long term (*p* < 0.05) (Table 1).

### 3.3. Risk Factors for SHV Occurrence

A multivariate logistic analysis was performed to analyze the risk factors for SHV occurrence. The variables were chosen based on the following points: (1) variables should be considered clinically relevant to SHV occurrence, (2) they showed a significant difference between the SHV group and the non-SHV group, and (3) considering the sample size, eight variables needed to be chosen to build a multivariable model for SHV occurrence.

We found that patients with the comorbidities of hypertension (OR 3.175, CI 1.332–7.569), postoperative usage of sedation drugs (OR 3.693, CI 1.563–8.724), and hypernatremia (OR 2.803, CI 1.070–7.340) had a positive correlation to SHV occurrence. However, patients who underwent hematoma removal treatment (OR 0.176, CI 0.068–0.460) had a significant, inverse association with SHV compared with patients who did not undergo hematoma removal treatment (Table 2).

### 3.4. ABG Results and Outcome

**Figure 2:**
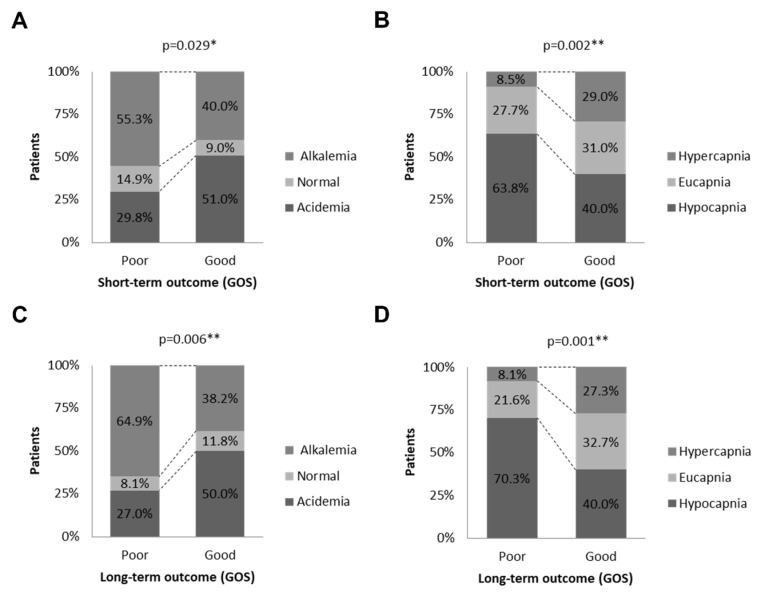
Neurologic outcome of patients was assessed by Glasgow Outcome Scale (GOS) score. (**A**,**C**), the association of acid–base balance with the short-term and long-term outcome. (**B**,**D**), the association of blood carbonic acid level with the short-term and long-term outcome. * *p* < 0.05, ** *p* < 0.01.

The association of the main parameters of ABG results with short-term and long-term outcomes was analyzed (Table 3). For short-term outcomes, 47 patients showed GOS scores of 1–3 when assessed at 2 weeks. The situation improved when assessed again at 6 months, with just 37 patients having a GOS score of 1–3. The pH values, which were divided into acidemia, normal, and alkalemia groups, showed significant differences in patients with poor outcomes and good outcomes in both the short term and long term (*p* < 0.05). This was also true for the PaCO_2_ values, which were divided into hypocapnia, eucapnia, and hypercapnia groups (Figure 2), and the HCO_3_^−^ values (Table 3).

**Figure 3:**
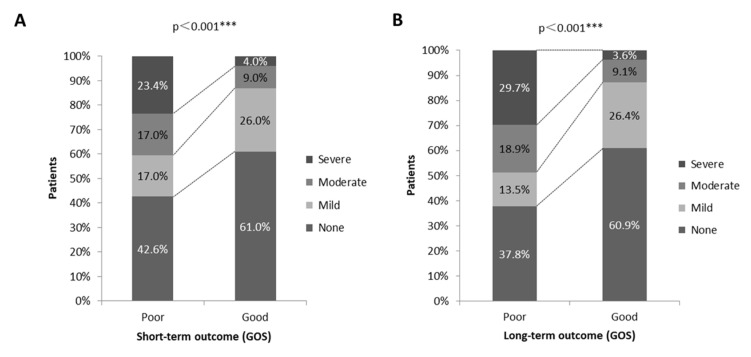
The association of SHV severity and neurologic outcome. Different severity of SHV had statistically significant difference between poor and good outcome for short-term period (**A**) and long-term period (**B**). *** *p* < 0.001.

When the ABG results were analyzed according to SHV severity, an interesting conclusion was drawn whereby SHV severity was associated with outcome (*p* < 0.001). In patients with poor short-term outcomes, 8(17.0%) patients had mild SHV, 8 (17.0%) patients had moderate SHV, and 11 (23.4%) patients had severe SHV. In patients with good short-term outcomes, 26 (26.0%) patients had mild SHV, 9 (9.0%) patients had moderate SHV, and 4 (4.0%) patients had severe SHV. In patients with long-term outcomes, the amounts were 5 (13.5%) patients, 7 (18.9%) patients, and 11 (39.7%) patients for poor outcomes and 29 (26.4%) patients, 10 (9.1%) patients, and 4 (3.6%) patients for good outcomes. SHV severity showed a significant difference in the poor and good outcome groups (Figure 3), especially severe SHV (*p* < 0.001) (Table 3).

## 4. Discussion

SHV was first identified in patients with acute brain stem disease [13]. Most reports of central hyperventilation refer to accounts of extreme hypocapnia in alert patients with brain tumors [10,11]. Hyperventilation is also a common manifestation of paroxysmal dysautonomia, a syndrome that also involves hypertension, tachycardia, fever, and dystonic posturing [26]. It has been proposed that structural disconnection of descending inhibitory pathways from the cortex, thalamus, or pons to the normal dorsal medullary respiratory centers by tumors could produce this syndrome. Our retrospective analysis of 147 patients with cerebellar hemorrhage showed a high incidence of SHV (approximately 44.9%). This incidence is consistent with the findings in other types of neurosurgical diseases. Notably, in subarachnoid hemorrhage (SAH), the frequency of SHV varies from 55% to 92% [8,9]. Similarly, the rate of SHV reported in severe TBIs is approximately 69%, including almost 33% for severe SHV [7,22]. The pathophysiology of this condition after a cerebellar hematoma has not been completely established, but multiple hypotheses can be discussed.

First, a cerebellar hematoma may induce SHV by compression of the brainstem, leading to dysfunction of the respiratory center. That is why, in our results, patients with SHV seem to have a larger volume of hematoma. Undoubtedly, abnormal respiratory function due to a hematoma at the onset of the disease plays an important role. Second, the increase in intracranial pressure caused by a cerebellar hematoma and secondary hydrocephalus causes brain ischemia and hypoxia, leading to an increase in lactic acid metabolism and hyperventilation. It has also been proposed that CNS lactate could cause hyperventilation by stimulating medullary chemoreceptors. This is supported by the presence of lactic acid in the CSF of a patient with SHV and diffuse cerebral lymphoma, with a decrease in lactic acid after treatment [27].

A GCS score < 6 was found to be an important factor in spontaneous hyperventilation, which is consistent with the discovery of Pierre Esnault et al. They found that patients with SHV had a more severe TBI in terms of median initial GCS score, rate of pupillary abnormality, and median head AIS score than those without SHV [22]. A total of 49.6% of patients had already been treated with antibiotics for more than 48 h before the onset of SHV. Similarly, SAH patients with spontaneous hyperventilation were significantly more likely to have higher mean WFNS and Hunt–Hess scores and experience pneumonia, neurogenic myocardial injury, and SIRS [9]. Thus, the duration of fever and hypernatremia were also significant factors in hyperventilation in the postoperative evaluation. A recent study of patients undergoing surgery for cerebellar hemorrhage revealed a postoperative ventilator rate of 85.5%, with 26.3% of patients remaining intubated beyond 10 days [28]. This finding suggests that respiratory recovery in cerebellar hemorrhage patients can be very slow. In addition, SHV could simply be due to other factors indirectly related to a cerebellar hematoma and its treatment, such as pain, discomfort from an endotracheal tube, fever due to an infection or analgesic, and sedative drug withdrawal syndrome. In fact, the duration of mechanical ventilation, usage of sedation drugs, and duration of fever all showed significant differences between the SHV group and the non-SHV group. As coma and mechanical ventilation are closely linked to the occurrence of SHV, the significant inverse association of hematoma removal is not surprising. Although the role of surgery for most patients with spontaneous ICH remains controversial, the American Heart Association (AHA) guidelines still recommend that patients with cerebellar hemorrhage who are deteriorating neurologically or who have brainstem compression and/or hydrocephalus from ventricular obstruction undergo surgical removal of the hemorrhage as soon as possible [20].

Another main finding of this study was the significant association between hypercapnia or hypocapnia and alkalemia or acidemia due to SHV and unfavorable functional neurologic outcome at two weeks and six months. In addition, the severity of SHV was also closely linked to both the short-term and long-term outcomes of patients with a cerebellar hematoma. However, the mechanism by which SHV affects the prognosis remains unclear. Many studies have found an adverse effect of hyperventilation in patients with a TBI, and these findings could help to explain unfavorable outcomes in cerebellar hemorrhage patients [4,8]. Hypocapnia is thought to cause or aggravate brain ischemia [4,29], while Coles et al. demonstrated that induced moderate hypocapnia (from 36 to 29 mm Hg) significantly reduces global CBF (from 31 to 23 mL/100 g/min) [30]. In addition, hypocapnia can induce the release of excitatory amino acids (N-methyl-d-aspartate and glutamate), increase neuronal excitability and glucose consumption, and potentiate and prolong convulsive activity [31,32]. Low levels of PaCO_2_ produce neurotoxic effects by inducing the release of cytotoxic excitatory amino acids, increasing dopamine levels in the basal ganglia, and promoting the incorporation of choline into the phospholipids of cell membranes [33]. Carrera et al. demonstrated that SHV may negatively affect brain tissue oxygenation monitored with brain tissue oxygen tension (PbtO_2_) in patients with a severe brain injury (SAH, intracranial hemorrhage, or a TBI), which also supports our finding [7].

Even though our study clarified an important clinical feature that had a significant association with the poor functional neurologic outcomes of patients with cerebellar hemorrhage, no evidence showed that correcting hyperventilation could improve the prognosis of a cerebellar hematoma. The opportunity for and methods of SHV treatment should also be taken into consideration. Thus, further investigation is still needed.

Several limitations should be considered when interpreting the results. First, due to the inclusion and exclusion criteria, sample selection bias may be present, leading to a bias in the results of the study. However, the design of the cohort study and the statistical analysis minimized the change in conclusions caused by this deviation. Second, the monitoring of hyperventilation or hypocapnia in this study was not performed dynamically or in real time, and the results were selected, but the duration of the situation and the sampling time had an impact on the patient’s test results. Third, the sample size of this study is limited, and our conclusions need to be further verified in a larger patient population.

## 5. Conclusions

In this retrospective analysis, we found that spontaneous hyperventilation was common and that its severity was associated with the functional outcome in cerebellar hemorrhage patients. As little attention has been paid to the manifestation of this clinical characteristic in spontaneous cerebellar hemorrhage patients, it is important to present our initial findings for further investigation and ultimately improve the prognosis of such patients.

## Figures and Tables

**Table 1:** Clinical characteristics of patients with and without spontaneous hyperventilation.

Variables	Total (*n* = 147)	SHV (*n* = 66)	Non-SHV (*n* = 81)	*p*-Value
Gender (Male)	87 (59.2%)	32 (48.5%)	55 (67.9%)	0.017 *
Age (years)	59.0 (50.0, 65.0)	58.0 (51.5, 64.3)	60.0 (48.0, 66.5)	0.764
Comorbidity				
Hypertension	85 (57.8%)	46 (69.7%)	39 (48.1%)	0.009 **
Diabetes Mellitus	23 (15.6%)	14 (21.2%)	9 (11.1%)	0.094
Heart dysfunction	13 (8.8%)	5 (7.6%)	8 (9.9%)	0.625
Lung dysfunction	15 (10.2%)	6 (9.1%)	9 (11.1%)	0.687
Others	18 (12.2%)	7 (10.6%)	11 (13.6%)	0.584
Hematoma				
Location (Unilateral/Middle)	121/26	54/12	67/14	0.887
Volume (mL)	18.1 ± 8.6	19.8 ± 8.3	16.8 ± 8.7	0.036 *
SAH	85 (57.8%)	34 (51.5%)	51 (63.0%)	0.162
Hydrocephalus	22 (15.0%)	9 (13.6%)	13 (16.0%)	0.683
GCS	8.0 (6.0, 15.0)	8.0 (5.0, 13.0)	10.0 (6.0, 15.0)	0.014 *
GCS < 6	28 (19.0%)	21 (31.8%)	7 (8.6%)	0.001 **
Time from onset to admission (hrs)	12.0 (7.0, 24.0)	12.0 (7.0, 24.0)	12.0 (6.0, 24.0)	0.724
Time from admission to surgery (hrs)	12.0 (6.0, 20.0)	10.0 (4.0, 20.3)	12.0 (7.0, 20.5)	0.126
Laboratory tests at admission				
Hypoproteinemia	23 (15.6%)	8 (12.1%)	15 (18.5%)	0.288
Hypernatremia	25 (17.0%)	14 (21.2%)	11 (13.6%)	0.221
Hypokalemia	28 (19.0%)	12 (18.2%)	16 (19.8%)	0.809
Hypocalcemia	13 (8.8%)	6 (9.1%)	7 (8.6%)	0.924
Hyperglycemia	124 (84.4%)	54 (81.8%)	70 (86.4%)	0.445
Anemia	54 (36.7%)	27 (40.9%)	27 (33.3%)	0.924
Treatment				
Hematoma removal	103 (70.1%)	38 (57.6%)	65 (80.2%)	0.003 **
Hematoma removal + DC	83 (56.5%)	36 (54.5%)	47 (58.0%)	0.672
EVD	64 (43.5%)	30 (45.5%)	34 (42.0%)	0.672
Tracheotomy	58 (39.5%)	25 (37.9%)	33 (40.7%)	0.724
MV	38 (25.9%)	11 (16.7%)	27 (33.3%)	0.023 *
Duration of MV (days)	3.5 (2.0, 4.5)	4.0 (3.0, 5.0)	3.2 (1.8, 4.1)	0.001 **
Usage of sedation drugs	81 (55.1%)	46 (69.7%)	35 (43.2%)	0.001 **
Duration of fever (days)	3.0 (2.0, 5.0)	3.5 (2.0, 6.0)	3.0 (1.0, 4.0)	0.033 *
Pneumonia	95 (64.6%)	40 (60.6%)	55 (67.9%)	0.358
Postoperative laboratory tests				
Hypoproteinemia	131 (89.1%)	56 (84.8%)	75 (92.6%)	0.134
Hypernatremia	44 (29.9%)	28 (42.4%)	16 (19.8%)	0.003 **
Hyponatremia	19 (12.9%)	9 (13.6%)	10 (12.3%)	0.817
Hypokalemia	66 (44.9%)	35 (53.0%)	31 (38.3%)	0.074
Hypocalcemia	113 (76.9%)	46 (69.7%)	67 (82.7%)	0.063
Hyperglycemia	123 (83.7%)	55 (83.3%)	68 (84.0%)	0.920
Anemia	105 (71.4%)	44 (66.7%)	61 (75.3%)	0.249
Short-term outcome				
Poor/Good	47/100	27/39	20/61	0.036 *
Long-term evaluation				
Poor/Good	37/110	24/42	13/68	0.005 **

* *p* < 0.05, ** *p* < 0.01. DC, decompressive craniectomy; EVD, external ventricular drainage; GCS, Glasgow Coma Scale; MV, mechanical ventilation; SAH, subarachnoid hemorrhage; SHV, spontaneous hyperventilation.

**Table 2:** The multivariate logistic analysis of risk factors for SHV occurrence.

Variables	B	Wald	OR	95% CI	*p*-Value
Gender (Male)	−0.686	2.491	0.504	0.215–1.180	0.114
Comorbidity (Hypertension)	1.155	6.791	3.175	1.332–7.569	0.009 **
Hematoma Volume (>18.1 mL)	−0.884	3.589	0.413	0.165–1.031	0.058
GCS (<6)	0.859	1.781	2.362	0.668–8.345	2.362
Hematoma removal	−1.736	12.578	0.176	0.068–0.460	<0.001 ***
MV	−0.911	3.472	0.402	0.154–1.048	0.062
Usage of sedation drugs	1.306	8.872	3.693	1.563–8.724	0.003 **
Hypernatremia	1.031	4.403	2.803	1.070–7.340	0.036 *

* *p* < 0.05, ** *p <* 0.01, and *** *p* < 0.001. SHV, spontaneous hyperventilation; B, beta; OR, odds ratio; GCS, Glasgow Coma Scale; MV, mechanical ventilation.

**Table 3:** The relationship between arterial blood gas test results and outcomes.

Variables	Short Term		*p*-Value	Long Term		*p*-Value
	Poor (*n* = 47)	Good (*n* = 100)		Poor (*n* = 37)	Good (*n* = 110)	
pH	7.46 (7.31, 7.53)	7.35 (7.28, 7.49)	0.055	7.48 (7.34, 7.56)	7.36 (7.28, 7.48)	0.015 *
pH group			0.029 *			0.006 **
Acidemia	14 (29.8%)	51 (51.0%)		10 (27.0%)	55 (50.0%)	
Normal	7 (14.9%)	9 (9.0%)		3 (8.1%)	13 (11.8%)	
Alkalemia	26 (55.3%)	40 (40.0%)		24 (64.9%)	42 (38.2%)	
PaCO_2_ (mmHg)	33.0 (26.0, 41.0)	39.0 (32.0, 48.8)	0.001 **	32.0 (23.5, 38.5)	39.0 (32.0, 48.0)	<0.001 ***
PaCO_2_ group			0.002 **			0.001 **
Hypocapnia	30 (63.8%)	40 (40.0%)		26 (70.3%)	44 (40.0%)	
Eucapnia	13 (27.7%)	31 (31.0%)		8 (21.6%)	36 (32.7%)	
Hypercapnia	4 (8.5%)	29 (29.0%)		3 (8.1%)	30 (27.3%)	
HCO_3_^−^ (mmol/L)	23.0 (19.2, 28.5)	27.3 (24.9, 30.5)	0.001 **	23.1 (19.7, 28.5)	27.1 (23.1, 30.5)	0.003 **
SHV severity			<0.001 ***			<0.001 ***
Mild	8 (17.0%)	26 (26.0%)	0.227	5 (13.5%)	29 (26.4%)	0.109
Moderate	8 (17.0%)	9 (9.0%)	0.174	7 (18.9%)	10 (9.1%)	0.136
Severe	11 (23.4%)	4 (4.0%)	<0.001 ***	11 (29.7%)	4 (3.6%)	<0.001 ***

* *p* < 0.05, ** *p* < 0.01, and *** *p* < 0.001. SHV, spontaneous hyperventilation.

## Data Availability

The data presented in this study are available on request from the corresponding author. The data are not publicly available for privacy reasons.
